# Gastrointestinal bypass surgery for non-strangulated adhesive small bowel obstruction

**DOI:** 10.1007/s00595-026-03352-7

**Published:** 2026-06-06

**Authors:** Yuta Yamamoto, Masato Kitazawa, Satoshi Nakamura, Satoru Miyazaki, Nao Hondo, Masahiro Kataoka, Hirokazu Tanaka, Tadaaki Shimizu, Naoki Ishizaka, Naoya Yamamoto, Yuji Soejima

**Affiliations:** Division of Gastroenterological, Hepato-Biliary-Pancreatic, Transplantation and Pediatric Surgery, Department of Surgery, Shinshu University School of Medicine, 3-1-1 Asahi, 390-8621 Matsumoto, Nagano, Japan

**Keywords:** Adhesive small bowel obstruction, Gastrointestinal bypass surgery, Conservative treatment, Recurrence

## Abstract

The effectiveness of gastrointestinal bypass surgery for treating non-strangulated adhesive small bowel obstruction (ASBO) remains unclear. We aimed to compare the outcomes of gastrointestinal bypass and conventional surgery in patients with non-strangulated ASBO who were unresponsive to conservative treatment. The patients (*n* = 55) were divided into a bypass group (*n* = 15) undergoing bypass surgery and a non-bypass group (*n* = 40) undergoing conventional surgery, including adhesiolysis or small bowel resection. The serum albumin levels and prognostic nutritional index were comparable between the two groups at 12 months postoperatively. No patients (0%) in the bypass group and 13 (32.5%) in the non-bypass group experienced recurrent ASBO after surgery (*p* = 0.008). The recurrence-free survival was significantly longer in the bypass group than in the non-bypass group (*p* = 0.030). Gastrointestinal bypass surgery may reduce long-term recurrence and thus represents a promising alternative surgical strategy for non-strangulated ASBO.

**Supplementary Information:**

The online version contains supplementary material available at https://doi.org/10.1007/s00595-026-03352-7.

## Introduction

Adhesive small bowel obstruction (ASBO) remains a common and challenging complication of abdominal surgery, accounting for a substantial proportion of hospital admissions for acute abdominal conditions [1, 2]. The initial management of non-strangulated ASBO is generally conservative and comprises bowel rest, nasogastric decompression, and fluid resuscitation. This strategy has been successful in many cases. However, a significant proportion of patients (17–59%) ultimately require surgical intervention due to treatment failure or recurrent episodes [3–6]. Operative management of ASBO is associated with a reduced risk of recurrence [7]. Although traditional surgical approaches, such as adhesiolysis and partial small bowel resection, are widely considered, they are associated with a considerable re-obstruction rate (10–30%) [8, 9].

Gastrointestinal bypass surgery has been used mainly for malignant bowel obstruction caused by peritoneal dissemination of advanced cancer, with the aim of avoiding high-risk dissection in hostile abdomens and providing durable symptom relief [10–12]. Despite its established role in malignant settings, the potential application of bypass surgery in patients with benign ASBO has not been well-defined. In selected patients with dense adhesions, multiple previous surgeries, or anatomically unfavorable conditions, bypass procedures may offer a less invasive and safer alternative to extensive adhesiolysis while effectively preventing further obstruction at the affected site. Therefore, we hypothesized that gastrointestinal bypass surgery could be an alternative operative strategy for managing ASBO. In this study, we aimed to evaluate the short- and long-term outcomes of bypass surgery compared with those of conventional surgical treatment for non-strangulated ASBO.

**Fig. 1:**
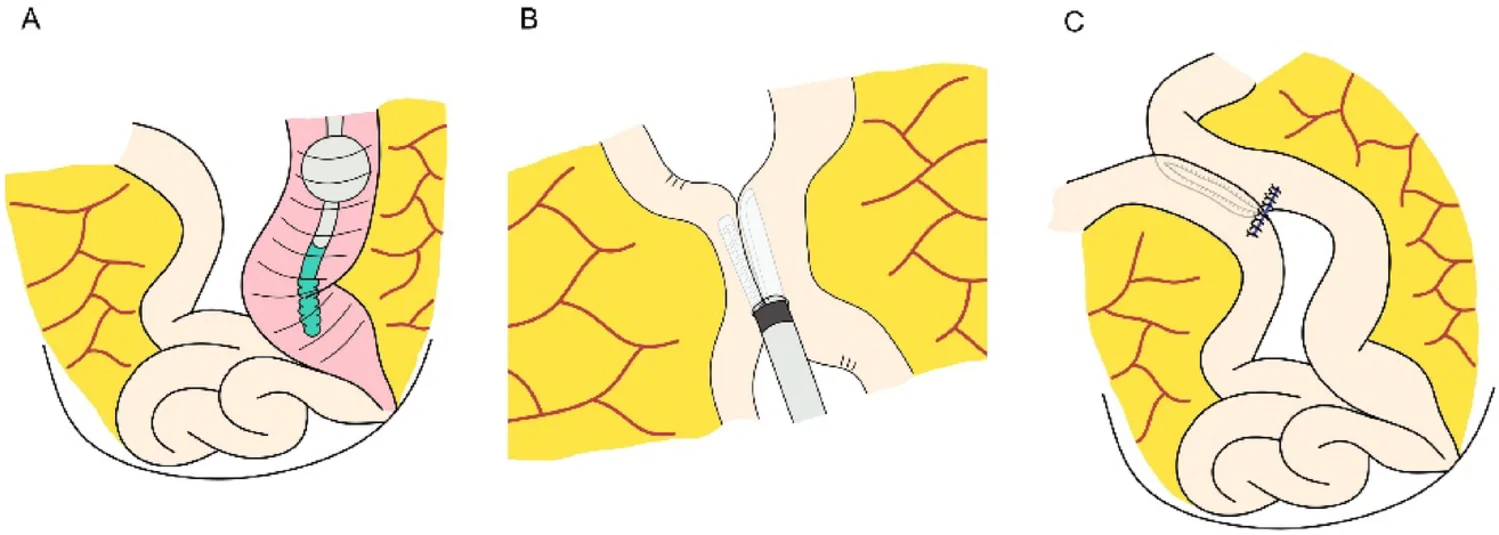
Bypass surgery for non-strangulated adhesive small bowel obstruction. (**A**) The long tube decompresses the bowel proximal (oral) to the site of obstruction. The bowel loops to be used for the bypass, both the proximal and distal (anal) sides, are identified. (**B**) A side-to-side anastomosis is created on the antimesenteric border with a 60-mm linear stapler. Because the lumen on the stapler entry side can become narrowed after closure of the enterotomy, the stapler is inserted through the enterotomy and direct it distally from the obstructed side. (**C**) The enterotomy is closed either with a 60-mm linear stapler or by hand-sewn suturing

## Methods

### Ethical consideration

This study was conducted in accordance with the ethical guidelines of the 2024 Declaration of Helsinki and was approved by the Institutional Ethical Committee of Shinshu University (approval number: 6616). This study was based on the ethical guidelines for clinical research in Japan and was approved by the Ethical Committee of Shinshu University Hospital. Informed consent was obtained from each patient included in this study using the opt-out method, and the details of the clinical research were published on the website of our institution, thus providing an opportunity for patients to refuse to participate in the study.

## Patients and study design

In this retrospective cohort study, we aimed to evaluate the effectiveness of gastrointestinal bypass surgery in patients with postoperative nonstrangulated ASBO admitted to Shinshu University Hospital between January 2011 and August 2024. ASBO was diagnosed based on clinical symptoms, including nausea, vomiting, and abdominal pain, as well as radiological imaging, which demonstrated a dilated small intestine with a diameter of > 2.5 cm. During the study period, 580 patients with postoperative ASBO unrelated to peritoneal dissemination of malignant tumors were admitted to our hospital. We excluded 447 patients who underwent successful conservative treatment and 78 who required emergency surgery for intestinal ischemia secondary to strangulation. Our final study group included 55 patients who underwent surgery for non-strangulated ASBO after failed conservative treatment. The patients were allocated into two groups: the bypass group (*n* = 15), which underwent various types of gastrointestinal bypass procedures, and the non-bypass group (*n* = 40), which underwent adhesiolysis alone or in combination with segmental small-bowel resection. A flow diagram of patient selection is presented in Online Resource 1.

**Fig. 2:**
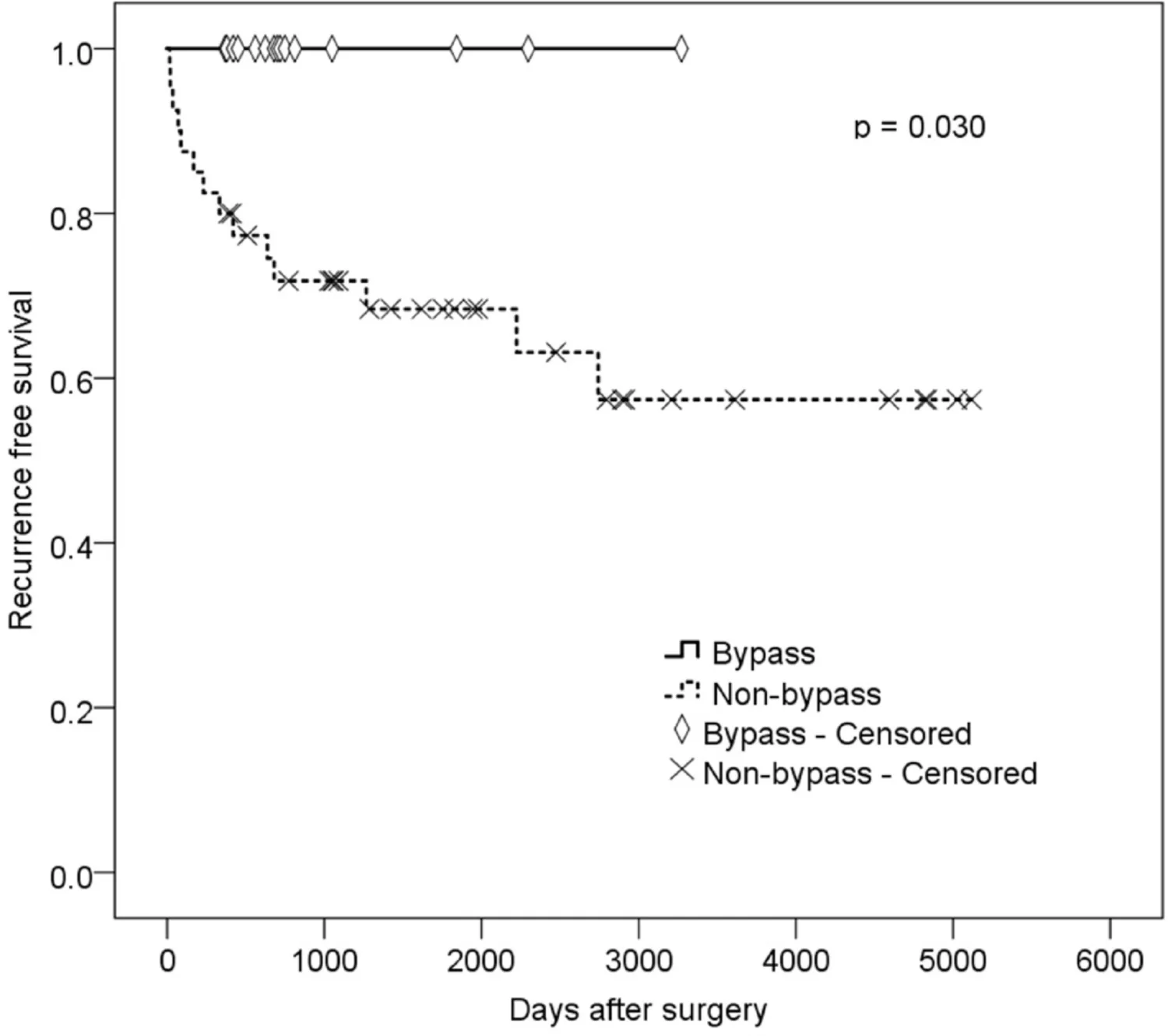
Proportion of the recurrence of adhesive small bowel obstruction among patients in the bypass group (solid line) and in the non-bypass group (dotted line). The rhombus and cross marks represent censoring in the bypass and non-bypass group, respectively

Preoperative, intraoperative, and postoperative variables, including demographics, operative details, nutritional status, and complications, were collected. The primary endpoint was recurrence-free survival. The secondary outcomes included operative time, blood loss, complications graded by Clavien–Dindo classification, serum albumin levels, prognostic nutritional index, and postoperative length of stay. Regarding managing ASBO, intravenous fluids were administered to all patients. Initially, we assessed the computed tomography findings of small intestine strangulation that required emergency surgery. When strangulation was excluded, the patients were considered candidates for conservative treatment, including nasogastric tube and long tube placement, to perform gastrointestinal decompression. When the obstructions persisted for more than 1 week or recurred after diet resumption, nonsurgical management was considered a failure, thereafter, the patients underwent surgery.

**Table 1:** Patient characteristics

Variables			Total (*n* = 55)
Female sex, n (%)			22 (41.8)
Age, median (IQR), years			72 (64–78)
Body mass index, median (IQR), kg/m^2^			18.6 (16.6–21.3)
Primary disease, n (%)			
	Benign		17 (30.9)
	Malignancy		38 (69.1)
		Colon/Rectum	17 (30.9)
		Esophagus/Stomach	8 (14.5)
		Uterus/Ovary	7 (12.7)
		Bladder/Urinary tract	5 (9.1)
		Other	1 (1.8)
Performance Status, n (%)			
		0	33 (60.0)
		1	15 (27.3)
		2	4 (7.3)
		3	3 (5.4)
		4	0 (0)
ASA-PS, n (%)			
		Ⅱ	41 (74.5)
		Ⅲ	13 (23.6)
		Ⅳ	1 (1.8)
Treatment, n (%)			
	Bypass surgery		15 (27.3)
		Stomach–Ileum	1 (1.8)
		Jejunum–Jejunum	2 (3.6)
		Jejunum–Transverse colon	1 (1.8)
		Ileum–Ileum	5 (9.1)
		Ileum–Ascending colon	1 (1.8)
		Ileum–Transverse colon	5 (9.1)
	Non-bypass surgery		40 (72.7)
		Only adhesiolysis	31 (56.4)
		Partial resection of small intestine	9 (16.4)

ASA-PS, American Society of Anesthesiologist Physical Score; BMI, body mass index; IQR, interquartile range

Postoperative follow-up was conducted through outpatient visits and a review of the medical records. ASBO recurrence was defined as readmission for bowel obstruction confirmed by clinical symptoms and radiological imaging. Patients were followed up until recurrence, death, or the last clinical visit. The nutritional status, including the serum albumin level and prognostic nutritional index, was evaluated at 6 months and 1 year after surgery in patients with available laboratory data.

**Table 2:** Comparisons of patients’ demographics between the bypass and non-bypass groups

		Bypass (*n* = 15)	Non-bypass (*n* = 40)	*p*-value
Female sex, *n* (%)		8 (53.3)	15 (37.5)	0.289
Age, median (IQR), years		71.0 (64.0–80.0)	72.5 (61.0–77.3)	0.684
BMI, median (IQR), kg/m^2^		16.7 (15.9–22.0)	19.3 (17.4–21.3)	0.249
Performance status 2-4, n (%)		4 (36.4)	3 (7.5)	0.079
ASA-PS ≥Ⅲ, n (%)		5 (33.3)	9 (22.5)	0.311
Primary disease, n (%)				
	Benign	3 (20.0)	14 (35.0)	0.233
	Esophagus/Stomach malignancy	2 (13.3)	6 (15.0)	0.624
	Colorectal malignancy	5 (33.3)	12 (30.0)	0.528
	Other malignancy	5 (33.3)	8 (20.0)	0.244
Surgical approach for primary disease, n (%)				
	Open	11 (63.6)	37 (92.5)	0.079
	Laparoscopy/Robot	4 (36.4)	3 (7.5)	
Medical history, n (%)				
	Cardiovascular disease	4 (36.4)	14 (35.0)	0.403
	Diabetes mellitus	0 (0)	5 (12.5)	0.189
	Renal Failure	2 (13.3)	2 (5.0)	0.298
	Neurological disease	2 (13.3)	7 (17.5)	0.532
	ASBO	5 (33.3)	21 (52.5)	0.205
Time to admission to surgery, median (IQR), days		13 (10–20)	12 (7–18)	0.368
Operative time, median (IQR), minutes		123 (96–144)	119 (94–161)	0.734
Blood loss, median (IQR), ml		25 (0–95)	50 (0–100)	0.570
Postoperative complications, n (%)				
	Clavien–Dindo grade ≥Ⅲ	5 (33.3)	5 (12.5)	0.086
	Clavien–Dindo all grades	8 (53.3)	14 (35.0)	0.216
Recurrence of ASBO after surgery, n (%)		0 (0)	13 (32.5)	0.008
Serum albumin, median (IQR), g/dl				
	Preoperative	3.2 (3.0–3.4)	3.3 (3.1–3.7)	0.023
	6 months postoperatively	3.7 (3.3–4.2)	4.1 (3.9–4.3)	0.046
	12 months postoperatively	4.0 (3.3–4.4)	4.0 (3.8–4.2)	0.560
Prognostic nutritional index, median (IQR)				
	Preoperative	36.9 (34.3–41.7)	38.0 (35.0–40.7)	0.030
	6 months postoperatively	48.6 (37.4–51.8)	46.5 (43.7–49.2)	0.674
	12 months postoperatively	50.4 (38.3–60.8)	46.0 (43.3–50.0)	0.514
Time to diet resumption, median (IQR), days		5 (3–8)	5 (4–10)	0.834
Postoperative length of stay, median (IQR), days		23 (13–31)	18 (12–23)	0.205
Follow-up period, median (IQR), days		701 (450–1049)	1277 (413–2783)	0.216

ASA-PS, American Society of Anesthesiologists Physical Status; ASBO, adhesive small bowel obstruction; BMI, body mass index; IQR, interquartile range

### Statistical analysis

Statistical analyses were performed using the Statistical Package for the Social Sciences (version 23.0; IBM Corp., Armonk, NY, USA). Demographic data are presented as descriptive statistics. Nonparametric data are presented as medians with interquartile ranges (IQRs). The Mann–Whitney U test was used to compare nonparametric data. Qualitative variables were compared using the chi-square test. Recurrence of ASBO-free survival was evaluated using the Kaplan–Meier analysis. All tests were two-tailed, and a *p*-value of < 0.05 was considered statistically significant.

## Surgical technique of gastrointestinal bypass surgery

The gastrointestinal bypass surgical technique used for non-strangulated ASBO is illustrated in Fig. 1. We routinely use a long intestinal decompression tube (“long tube”) as part of the conservative management of ASBO; consequently, a long tube is in place at the time of surgery. The tube decompresses the bowel proximal (oral) to the site of obstruction, thereby improving the bowel condition and reducing anatomical misidentification because an intraluminal long tube indicates the proximal side of the bowel.

During surgery, we first identified the obstructed site or intestine proximal to the site, guided by the tactile feedback from the intraluminal tube. We then identified the bowel loops to be used for bypass on both the proximal and distal (anal) sides of the patient. As noted above, the long intraluminal tube is a useful marker for confirming the proximal side. To avoid interference with the bypass, the long tube was withdrawn by several tens of centimeters from the patient. A side-to-side anastomosis was performed on the antimesenteric border using a 60-mm linear stapler. Because the lumen on the stapler entry side could narrow after closure of the enterotomy, we inserted the stapler through the enterotomy and directed it distally from the obstructed side. The enterotomy was closed using either a 60-mm linear stapler or hand-sewn suturing. When small intestine–small intestine bypass was not feasible, then the stomach or colon was bypassed by the small intestine.

## Results

The baseline patient characteristics are presented in Table 1. The majority of patients had visceral malignancies as the primary indication for previous abdominal surgery. Seven patients (12.7%) had an impaired performance status (PS) (ECOG PS 2 or 3). Regarding bypass surgery, ileum-ileum and ileum-transverse bypass were the most frequent (five cases each [9.1%]). Bypass surgery using the stomach was performed in only one patient (1.8%). Partial resection of the small intestine was performed in nine patients (16.4% of total patients).

There were no significant differences in the patients’ baseline demographics, except for nutritional status, between the two groups (Table 2). The patients in the bypass group showed lower preoperative serum albumin levels and prognostic nutritional indices than did those in the non-bypass group (3.2 [3.0–3.4] vs. 3.3 [3.1–3.7] g/dl, *p* = 0.023 and 36.9 [34.3–41.7] vs. 38.0 [35.0–40.7], *p* = 0.030, respectively). However, at 12 months postoperatively, serum albumin levels and prognostic nutritional indices became comparable between the two groups (4.0 [3.3–4.4] vs. 4.0 [3.8–4.2] g/dl, *p* = 0.560, and 50.4 [34.3–41.7] vs. 46.0 [43.3–50.0], *p* = 0.514, respectively). The operative time, blood loss, postoperative complications, and postoperative length of hospital stay were comparable between the two groups. The follow-up period was 701 (450–1049) days in the bypass group and 1277 (413–2783) days in the non-bypass group (*p* = 0.216). No patients (0%) in the bypass group and 13 (32.5%) in the non-bypass group experienced recurrent ASBO after surgery (*p* = 0.008). Using the Kaplan–Meier survival analysis, the recurrence-free survival significantly improved in the bypass group compared to the non-bypass group (*p* = 0.030) (shown in Fig. 2). Online resources 2–5 show the representative findings from a single case, including a schematic illustration of the bypass procedure and pre- and postoperative CT images.

## Discussion

The present study revealed that bypass surgery could reduce ASBO recurrence in patients in whom conservative treatment failed. In addition, the long-term nutritional outcomes were comparable. These findings support the utility of bypass surgery not only in select cases where dense adhesions or repeated obstructions preclude conventional approaches, but also as a promising surgical option in patients eligible for conventional adhesiolysis or partial small bowel resection.

No cases of recurrent ASBO were observed in patients who underwent bypass surgery. Bypass was selected for treating cases wherein conventional adhesiolysis or segmental small bowel resection was considered difficult, such as those with complex adhesions, dense/fibrotic adhesions, or adhesions located deep within the pelvis. The finding that these patients experienced fewer ASBO recurrences was contrary to our expectations. We avoided unnecessary adhesiolysis during operations for ASBO, as excessive adhesiolysis could further injure the serosa, provoke inflammation leading to postoperative ileus, and ultimately predispose the patient to new adhesions [13–15].

Since the small intestine is responsible for nutrient absorption, shortening of the small bowel could compromise the nutritional status [16, 17]. Accordingly, preserving small bowel length and preventing short bowel syndrome are key considerations in intestinal surgery [18]. When a gastrointestinal bypass is performed and luminal contents traverse the bypassed part, the effective length of the small intestine available for absorption is shortened, potentially leading to a nutritional decline [19]. Nevertheless, in the present study, the postoperative nutritional indices were comparable between the two groups. Although this was not verified by contrast gastrointestinal imaging, it is possible that even after a bypass procedure, the luminal contents may partially pass through the native intestinal tract once edema at the obstructed segment subsides and the narrowing reduces. However, this mechanism remains speculative and it was not directly evaluated in this study. We consider that avoiding unnecessary adhesiolysis, together with the availability of two potential routes for luminal transit, could account for the low recurrence rate of ASBO after gastrointestinal bypass and finding that the postoperative nutritional status was comparable to that after conventional surgery for ASBO. Although a systematic imaging analysis was not performed in this study, postoperative CT images demonstrated a reduction in bowel dilatation and fluid accumulation around the previously obstructed segment after bypass surgery (Online Resources 2–4). In addition, a small amount of intestinal fluid was observed in the ileum distal to the previously obstructed site which suggested the passage of luminal contents through the site. These findings support the hypothesis that decompression through the bypass route can facilitate resolution of edema and luminal narrowing at the obstructed site.

In the present study, several types of bypass procedures were performed depending on intraoperative findings (Table 1). Although the specific anastomotic configurations differed, the underlying surgical concept was consistent across the procedures. The primary goal of bypass surgery in this context is to avoid extensive adhesiolysis in hostile abdomens and restore intestinal continuity by bypassing the obstructed segment. Therefore, the favorable outcomes observed in this study may reflect the effectiveness of this surgical strategy rather than the superiority of a specific bypass technique. However, the heterogeneity of bypass procedures should be considered when interpreting the generalizability of our findings.

Anastomotic strictures are common complications of gastrointestinal surgery. Since linear staplers are associated with a lower stricture rate [19, 20], we used a linear stapler to create a side-to-side anastomosis during bypass. In addition, because closure of the common enterotomy can narrow the bowel lumen, we inserted a linear stapler from the limb on the obstructed side and fired it in the direction away from the obstruction.

To the best of our knowledge, this study is the first to statistically evaluate the efficacy of gastrointestinal bypass surgery in non-strangulated ASBO. Although the sample size was limited, the absence of recurrence in the bypass group and the significantly prolonged time to recurrence are important for managing patients with ASBO. The absence of any deterioration in the nutritional status in the bypass group suggests that our selection of bowel segments for anastomosis and the anastomotic technique were appropriate, and that the maintenance of two potential pathways for luminal transit is effective for nutrient absorption.

This study has several limitations. First, it was a retrospective study conducted at a single institution, and the choice of surgical procedures was not standardized. The decision to perform bypass surgery was made preoperatively and intraoperatively based on factors such as the extent and location of adhesions, feasibility of adhesiolysis, and overall condition of the bowel. Therefore, surgeon preference and experience may have influenced the selection of the surgical approach, thus introducing some potential selection bias. Second, the number of patients who underwent bypass surgery was low. Although our results suggest a potential benefit of bypass surgery in terms of reducing ASBO recurrence without compromising the nutritional status, the limited sample size restricts the statistical robustness of these findings. Third, the proposed mechanism explaining the nutritional status preservation after bypass surgery remains largely hypothetical because systematic imaging or physiological assessments were not performed in this study. Although representative postoperative CT findings may support our hypothesis (Online Resource 4), these observations were not analyzed in a standardized way. Future studies incorporating systematic imaging analyses may help to clarify the physiological mechanisms underlying these observations.

Despite these limitations, this study provides the first statistical comparison of gastrointestinal bypass surgery and conventional surgical treatment for non-strangulated ASBO. The absence of recurrence in the bypass group and the significantly prolonged recurrence-free survival observed in this study suggest that bypass surgery is a valuable alternative surgical option for selected patients.

In conclusion, gastrointestinal bypass surgery may represent a promising alternative surgical strategy for refractory non-strangulated ASBO, with a superior recurrence-free survival and comparable long-term nutritional outcomes. Patient selection and surgical expertise are crucial for optimizing outcomes. Further large-scale studies are required to confirm these findings.

## Electronic Supplementary Material

Below is the link to the electronic supplementary material.

Supplementary Material 1

Supplementary Material 2

Supplementary Material 3

Supplementary Material 4

## References

[1] Ten Broek RPG, Krielen P, Di Saverio S, Coccolini F, Biffl WL, Ansaloni L, et al. Bologna guidelines for diagnosis and management of adhesive small bowel obstruction (ASBO): 2017 update of the evidence-based guidelines from the world society of emergency surgery ASBO working group. World J Emerg Surg. 2018;13:24. 29946347 10.1186/s13017-018-0185-2 PMC6006983

[2] Jackson P, Vigiola Cruz M. Intestinal obstruction: Evaluation and management. Am Fam Physician. 2018;98:362–7. 30215917

[3] Maienza E, Godiris-Petit G, Noullet S, Menegaux F, Chereau N. Management of adhesive small bowel obstruction: The results of a large retrospective study. Int J Colorectal Dis. 2023;38:224. 37668744 10.1007/s00384-023-04512-8 PMC10480247

[4] Tong JWV, Lingam P, Shelat VG. Adhesive small bowel obstruction—An update. Acute Med Surg. 2020;7:e587. 33173587 10.1002/ams2.587 PMC7642618

[5] Mortensen MR, Alouda M, Bond Z, Burcharth J, Finne KF, Jensen TK, et al. One-year outcomes following operative or non-operative management of adhesional small bowel obstruction. BJS Open. 2023;7:zrad103. 37837353 10.1093/bjsopen/zrad103 PMC10576245

[6] Yamamoto Y, Kitazawa M, Otsubo T, Miyagawa Y, Tokumaru S, Nakamura S, et al. Impact of seasonal and meteorological factors on the incidence of adhesive small bowel obstruction: A large-scale study using a national inpatient database. Ann Gastroenterol Surg. 2022;6:569–76. 35847441 10.1002/ags3.12541 PMC9271017

[7] Behman R, Nathens AB, Mason S, Byrne JP, Hong NL, Pechlivanoglou P, et al. Association of surgical intervention for adhesive small-bowel obstruction with the risk of recurrence. JAMA Surg. 2019;154:413–20. 30698610 10.1001/jamasurg.2018.5248 PMC6537786

[8] Lorentzen L, Øines MN, Oma E, Jensen KK, Jorgensen LN. Recurrence after operative treatment of adhesive small-bowel obstruction. J Gastrointest Surg. 2018;22:329–34. 29030779 10.1007/s11605-017-3604-x

[9] Duron JJ, Silva NJD, du Montcel ST, Berger A, Muscari F, Hennet H, et al. Adhesive postoperative small bowel obstruction: Incidence and risk factors of recurrence after surgical treatment: A multicenter prospective study. Ann Surg. 2006;244:750–7. 17060768 10.1097/01.sla.0000225097.60142.68 PMC1856591

[10] Ferguson HJM, Ferguson CI, Speakman J, Ismail T. Management of intestinal obstruction in advanced malignancy. Ann Med Surg (Lond). 2015;4:264–70. 26288731 10.1016/j.amsu.2015.07.018 PMC4539185

[11] Ito Y, Fujitani K, Sakamaki K, Ando M, Kawabata R, Tanizawa Y, et al. QOL assessment after palliative surgery for malignant bowel obstruction caused by peritoneal dissemination of gastric cancer: A prospective multicenter observational study. Gastric Cancer. 2021;24:1131–9. 33791885 10.1007/s10120-021-01179-4

[12] Neri B, Citterio N, Schiavone SC, Biasutto D, Rea R, Martino M, et al. Malignant bowel occlusion: An update on current available treatments. Cancers (Basel). 2025;17:1522. 40361449 10.3390/cancers17091522 PMC12071143

[13] Moris D, Chakedis J, Rahnemai-Azar AA, Wilson A, Hennessy MM, Athanasiou A, et al. Postoperative abdominal adhesions: Clinical significance and advances in prevention and management. J Gastrointest Surg. 2017;21:1713–22. 28685387 10.1007/s11605-017-3488-9

[14] Sturm MCK, Abazid A, Stope MB. Tissue adhesion after surgical interventions (Review). Exp Ther Med. 2025;29:97. 40165802 10.3892/etm.2025.12847 PMC11956145

[15] Liao J, Li X, Fan Y. Prevention strategies of postoperative adhesion in soft tissues by applying biomaterials: Based on the mechanisms of occurrence and development of adhesions. Bioact Mater. 2023;26:387–12. 36969107 10.1016/j.bioactmat.2023.02.026 PMC10030827

[16] Nightingale J, Woodward JM. Small Bowel and Nutrition Committee of the British Society of Gastroenterology. Guidelines for management of patients with a short bowel. Gut. 2006;55:iv1–i12. 16837533 10.1136/gut.2006.091108 PMC2806687

[17] Massironi S, Cavalcoli F, Rausa E, Invernizzi P, Braga M, Vecchi M. Understanding short bowel syndrome: Current status and future perspectives. Dig Liver Dis. 2020;52:253–61. 31892505 10.1016/j.dld.2019.11.013

[18] Iyer K, DiBaise JK, Rubio-Tapia A. AGA clinical practice update on management of short bowel syndrome: Expert review. Clin Gastroenterol Hepatol. 2022;20:2185–e942. 35700884 10.1016/j.cgh.2022.05.032

[19] Chang KK, Patel MS, Yoon SS. Linear-stapled side-to-side esophagojejunostomy with hand-sewn closure of the common enterotomy after prophylactic and therapeutic total gastrectomy. J Gastrointest Surg. 2017;21:712–22. 27882512 10.1007/s11605-016-3326-5 PMC5360481

[20] Huang H, Guo Z, Li W, Zhang M, Li Y. A comparative study of using linear anastomosis with circular anastomosis in digestive tract reconstruction after laparoscopic radical total gastrectomy: A retrospective study. Med (Baltim). 2023;102:e34588. 10.1097/MD.0000000000034588 PMC10476817 37657064

