# Supplementary material for: Gastrointestinal bypass surgery for non-strangulated adhesive small bowel obstruction
Source: Surg Today. 2026 Jun 6;56(10):2543–9. doi: 10.1007/s00595-026-03352-7 (PMC13558432; doi:10.1007/s00595-026-03352-7)
Supplement: Supplementary file 1 [file 595_2026_3352_MOESM1_ESM.docx]

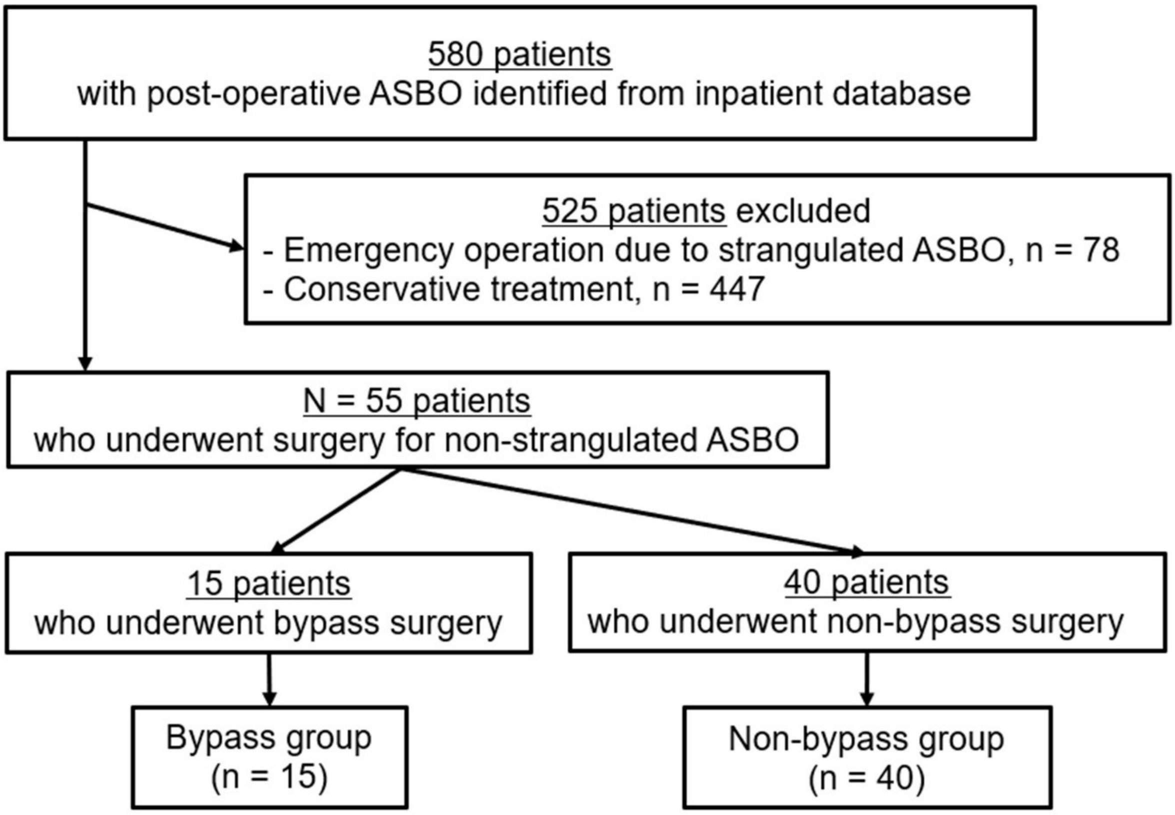
580 patients
with post-operative ASBO identified from inpatient database
525 patients excluded
- Emergency operation due to strangulated ASBO, n = 78
- Conservative treatment, n = 447
N = 55 patients
who underwent surgery for non-strangulated ASBO
15 patients
who underwent bypass surgery
40 patients
who underwent non-bypass surgery
Bypass group
(n = 15)
Non-bypass group
(n = 40)

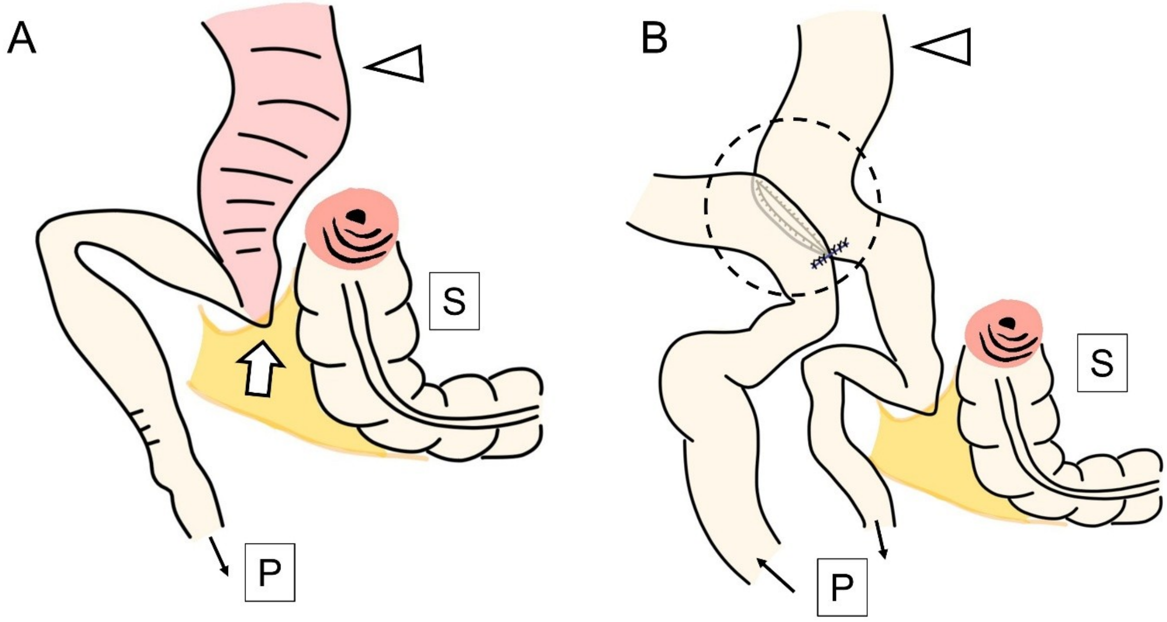
A
S
P
B
S
P
