# Supplementary material for: Gastrointestinal bypass surgery for non-strangulated adhesive small bowel obstruction
Source: Surg Today. 2026 Jun 6;56(10):2543–9. doi: 10.1007/s00595-026-03352-7 (PMC13558432; doi:10.1007/s00595-026-03352-7)
Supplement: Supplementary file 2 [file 595_2026_3352_MOESM4_ESM.docx]

**Figure legends for Online Resources**

**Online Resource 1.**

Flow diagram of patient selection.

**Online Resource 2.**

Schematic illustration of preoperative (A) and postoperative condition (B) after ileum–ileum bypass for adhesive small bowel obstruction. White arrowheads indicate the ileum proximal to the obstructed site. A white arrow indicates the site of adhesion causing obstruction, where the ileum is adherent to the mesentery of a sigmoid colostomy. The dotted circle indicates the bypass site. S, sigmoid colostomy; P, pelvic cavity.

**Online Resource 3.**

Contrast-enhanced CT images obtained during adhesive small bowel obstruction. The ileum adherent to the mesentery of the sigmoid colectomy represents the transition point of the obstruction, with dilatation of the proximal small bowel. The ileum in the pelvic cavity shows reduced intraluminal contents.

**Online Resource 4.**

Non-contrast CT images obtained 18 months after ileum–ileum bypass for adhesive small bowel obstruction. Dilatation of the small bowel, including the segment proximal to the bypass site, has improved. A small amount of intraluminal contents is observed in the ileum within the pelvic cavity distal to the previously obstructed site, suggesting a potential contribution of the bypass to both proximal decompression and improvement of the prior obstructive segment.
